# A Comparison of Postpartum Depression in Mothers Conceived by Assisted Reproductive Technology and Those Naturally Conceived

**DOI:** 10.22074/ijfs.2020.5466

**Published:** 2019-11-11

**Authors:** Elham Amirchaghmaghi, Farideh Malekzadeh, Mohammad Chehrazi, Zahra Ezabadi, Shokufeh Sabeti

**Affiliations:** 1 Department of Regenerative Biomedicine, Cell Science Research Center, Royan Institute for Stem Cell Biology and Technology, ACECR, Tehran, Iran; 2 Department of Endocrinology and Female Infertility, Reproductive Biomedicine Research Center, Royan Institute for Reproductive Biomedicine, ACECR, Tehran, Iran; 3 Reproductive Epidemiology Research Center, Royan Institute for Reproductive Biomedicine, ACECR, Tehran, Iran; 4 Department of Biostatistics and Epidemiology, School of Medicine, Babol University of Medical Sciences, Babol, Iran; 5 Department of Medical Education, School of Medicine and Center for Educational Research in Medical Sciences (CERMS), Iran University of Medical Sciences, Tehran, Iran

**Keywords:** Assisted Reproductive Technology, Edinburgh Postnatal Depression Scale, Natural Pregnancy, Postpartum Depression

## Abstract

**Background:**

It is thought that mothers who conceive via assisted reproductive technology (ART) may be at greater risk of postpartum depression (PPD) because of the problems and psychological stresses associated with ART treat- ment. The aim of the present study is to determine the occurrence of PPD among mothers who conceive by ART in comparison with mothers who naturally conceive. The Edinburgh Postnatal Depression Scale (EPDS) was used to assess PPD.

**Materials and Methods:**

This historical cohort study investigated 406 mothers with infants aged 3-9 months. Three hundred and eight women with natural pregnancies were selected as the control group from mothers who referred to Tehran healthcare centres for infant vaccinations. The ART group consisted of 98 women who conceived via ART at Royan Institute. Participants completed a general questionnaire that asked about education, occupation, number of children, delivery method, history of infant hospitalization, breastfeeding, mothers’ and infants’ ages, cause of infertil- ity (ART group), and history of depression. A validated Persian version of the EPDS was used to measure depressive symptoms.

**Results:**

The mean EPDS score in mothers who naturally conceived was 8.38 ± 0.35 in comparison with mothers who conceived via ART (7.59 ± 0.63). The proportions of women who reported PPD were 26.0% for the control group and 20.4% for the ART group. There was no statistically significant difference in PPD between the control and ART groups (P=0.26).

**Conclusion:**

The occurrence of PPD in mothers who conceived via ART was similar to those who conceived naturally.

## Introduction

Pregnancy is a welcomed event, usually associated with psychological and behavioral changes, especially for women who have become pregnant by an assisted reproductive technology (ART) (1). Postpartum depression (PPD) is important because it reduces the ability of a mother to care for her infant and decreases the quality of the relationship between the mother and her infant (2). Moreover, it increases the risk of future depression for the mother, and could negatively affect the mother and child relationship (3). Kettunen et al. (4) reported that infants having symptoms and illnesses, especially from infantile colic, were more common among depressed than nondepressed mothers.

The prevalence of PPD has been reported as 10-15% in different countries; however, in a systematic review carried out by Halbreich and Karkun, the prevalence of PPD was 0-60% in 40 countries (5). In two Iranian cities (Tabriz and Bousher) this value was determined by the Edinburgh Postnatal Depression Scale (EPDS), with estimates of 34.7 and 15.5% as reported by Sehhatie Shafaei et al. (6) and Bagherzadeh et al. (7), respectively.

In recent years, the impact of infertility on the mental health of couples has been more widely considered. Of note, infertility is a very unpleasant experience for many couples (8). Seibel and Taymor have reported that the frequency of psychological problems is 20- 25% in infertile couples (9). The experience of using fertility methods is unpleasant and difficult for both couples, but it is harder for women because they must take most of the medications (10). Moreover, infertility treatments usually cause high levels of stress for couples (11). It has been shown that women who undergo ART experience anxiety and depression (12). Depression, anxiety, and health problems are common reactions at the time of medical treatments in infertile couples, with estimates of 19.1% in women and 14.6% in men. Multiple failures in these treatments can negatively affect self-esteem and increase depression symptoms in infertile women (11).

 Studies have shown that mothers who conceive via ART are more emotionally vulnerable and have higher levels of distress compared to those who conceive naturally (13, 14). Fisher and colleagues have reported that those who conceive via ART show significantly more early parenting difficulties, which can negatively affect interactions between the mother and her infant. They conclude that these mothers need more support during pregnancy and after birth (15).

Based our searches, there has been no study in Iran that compared PPD in mothers who conceived via ART with those who conceived naturally. Therefore, we designed this study to use the EPDS to compare the frequency of PPD and its risk factors among these groups.

## Materials and Methods

This historical cohort study investigated 406 mothers of 3-9-month-old infants. We used convenience sampling methods for patient selection. The control group consisted of 308 mothers who had natural pregnancies and referred to the health centers affiliated with three main medical universities in Tehran, Iran for child vaccinations. The ART group consisted of mothers with 3-9-month-old infants convinced by ART and selected from the registry data bank at Royan Institute, Tehran, Iran. From these, we selected 98 mothers as the ART group.

The study was approved by the Ethics Committee of Royan Institute. All mothers signed a consent form before completing the questionnaire. The questionnaire was completed by each of the control group mothers. The ART mothers were contacted by telephone to complete the questionnaire. Questions that pertained to the mothers’ and infants’ ages, education, occupation, number of children, delivery method, history of infant hospitalization, breastfeeding, causes of infertility in women with infertility issues, and history of depression, along with the EPDS, were answered by each mother.

The EPDS is one of the most important screening tools used to identify PPD. It is a short, 10-item questionnaire that has a score from 0 to 30. Questions 1, 2, and 4 are scored from 0 to 3, whereas questions 3 and 5-10 are scored from 3 to 0 are scored (16). Although it was developed for English-speaking populations, the EPDS has been validated in non-English populations. Montazeri et al. (16) validated the Persian version of the EPDS in an Iranian population and reported that the questionnaire was acceptable, reliable, and valid for this population with a Cronbach's alpha coefficient of 0.86 and test-rest reliability (interclass correlation coefficient) of 0.80. Based on the EPDS scores, we categorized the mothers into two groups according to their scores: non-depressed (score: 0-9) and depressed (score: ≥10). Mothers with total scores of ≥10 should be further examined for depression (17).

The sample size was calculated based on at least 4% of the clinical differences of 2.6% depressed for the ART group and 6.7% depressed for the control group in EPDS between the ART and control groups. In order to determine the sample size, the power to detect the difference and type one error were set to 0.8 and 0.05, respectively.

Data analysis was performed using the Statistical Package for the Social Sciences (SPSS) software, version 20. Results were presented as proportions (percentages) and mean ± standard error (SE) or standard deviation (SD). One-way ANOVA, followed by Tukey and Dennett’s tests for multiple comparisons, were used to compare depression scores among education levels, causes of infertility, numbers of pregnancies and breastfeedings. The chi-square test was applied to compare the numbers of depressed individuals between the control and ART groups. The t test was used to compare continuous variables between normal and depressed samples. P<0.05 were considered statistically significant.

## Results

The mean age of the mothers was 28.87 ± 5.18 years (range: 17-51 years) and the infants had a mean age of 5.37 ± 1.30 months (range: 3-9 months). The percentage of mothers who reported PPD were 26.0% in the control group and 20.4% in the ART group, which was not statistically significant (P=0.26).

The mean ± SE score for EPDS in mothers who conceived naturally was 8.38 ± 0.35 and it was 7.59 ± 0.63 for mothers who conceived via ART. The difference was not statistically significant (P=0.27).

Our results showed that among mothers who conceived naturally, education level, occupation, and history of depression were significantly related to PPD. However, among mothers in the ART group, the type of delivery, history of infant’s hospitalization, and history of depression had a statistical correlation with PPD (Table 1).

**Table 1:** Comparison between the studied variables and PPD scores in the control and ART groups


Variables		Naturally conceived	ART conceived

Education		P=0.01^*^	P=0.12
	Less than diploma (n=58)	9.98 (0.88)	6.92 (1.09)
	Diploma (n=131)	8.79 (0.53)	9.12 (1.04)
	Higher than diploma (n=119)^a^	7.15 (0.52)	6.30 (1.06)
Occupation		P<0.001^*^	P=0.59
	Employed (n=71)	5.69 (0.64)	6.95 (1.45)
	Housewife (n=236)	9.14 (0.39)	7.76 (0.69)
Type of delivery		P=0.21	P=0.016^*^
	Vaginal (n=102)	7.73 (0.59)	12.33 (3.84)
	Cesarean (n=205)	8.67 (0.43)	7.44 (0.52)
History of infant hospitalization		P=0.21	P=0.016^*^
	No (n=96)	9.04 (0.60)	10.03 (1.32)
	Yes (n=210)	8.09 (0.43)	6.35 (0.61)
History of depression		P=0.015^*^	P=0.03^*^
	No (n=15)	12.20 (1.63)	12.12 (1.23)
	Yes (n=287)	8.21 (0.36)	7.18 (0.65)
Number of last pregnancies		P=0.99	P=0.56
	One (n=277)	8.33 (0.36)	8.02 (0.77)
	Two (n=18)	8.27 (1.50)	6.45 (1.08)
	Three or more (n=2)	8.50 (6.50)	6.85 (2.72)
Number of children		P=0.36	P=0.13
	One (n=172)	8.57 (0.47)	8.19 (0.79)
	Two or more (n=131)	7.92 (0.53)	6.13 (1.0)
Baby feeding status		P=0.17	P=0.49
	Breastfeeding (n=181)	8.95 (0.44)	6.93 (0.87)
	Breastfeeding plus milk powder (n=91)	7.78 (0.70)	8.76 (1.37)
	Milk powder (n=34)	7.29 (1.0)	7.53 (1.16)


*; Significant at P<0.05, ^a^; Significant between groups with education levels of less than diploma and higher than diploma, SE; Standard error, ART; Assisted reproductive technology, and PPD; Postpartum depression. Data are presented as mean ± SE.

Our results also showed that causes of infertility were

**Table 2:** Comparison of PPD scores and causes of infertility


Causes of infertility	n (%)	Mean score(± SE)	P value

Male factor	57 (58.2)	8.31 (0.86)	0.26
Female factor	19 (19.4)	4.73 (1.26)	
Both (male and female) factors	14 (14.3)	7.07 (1.24)	
Unexplained	8 (8.1)	10.12 (2.36)	


SE; Standard error and PPD; Postpartum depression.

## Discussion

We evaluated the occurrence of PPD among mothers who conceived via ART in comparison with those who conceived naturally due to the impact of mothers’ PPD in caring for their infants. In our study, the occurrence of PPD was 20.4% in the ART group and 26% in the control group. These results were consistent with other studies in Iran (16-18). Montazeri et al. (16) reported 22% of women with PPD at 6-8 weeks and 18% at 12-14 weeks after childbirth. Another Iranian study reported the level of PPD in women at 30% (18).

About 13-19% of mothers who have recently given birth experience depression during the postpartum period (19). These differences in the numbers of women with PPD are probably due to differences in body mass index (20), age (21), race/ethnicity (22), cultural, social and economic status, mental health perceptions, and other environmental factors (poverty, social support or perception, nutrition, stress, and biological vulnerability) (5).

PPD is a multidimensional disorder. The determination of factors (biological, psychological, and social) that predispose a mother to PPD will help identify at-risk mothers (23). One of the factors that has been suggested to increase PPD is infertility. However, in the literature, we have found three meta-analyses on PPD and none reported pregnancy via ART as a potential risk factor for PPD (24- 26). In a systematic review, Ross et al. (27) showed that the risk of a higher prevalence of PPD in mothers who become pregnant via ART was very low or unchanged in comparison to those with natural pregnancies. It seemed that women who have conceived through ART usually have a more intense emotional attachment to the fetus than women with spontaneous pregnancies (28). Although the mean EPDS score in mothers who conceived naturally was slightly higher than those who conceived via ART, we found no significant difference between PPD in both groups. However, Monti et al. (29) reported that the average PPD scores in mothers who conceived via ART were higher than those who conceived naturally, but the difference between the two groups was not statistically significant by using a cut-point of more than 12. This finding was similar to those reported by Chatziandreou et al. (30) and Listijono et al. (23). The results of metaanalyses by Gressier et al. (31) showed no increased risk of significant post-partum depressive symptoms after medically assisted conception.

Our study showed a significant relationship between PPD and type of delivery in mothers who conceived by ART, which did not agree with the results reported by Sadr et al. (34). These findings supported the results of a study by Rahmani et al. (36). Kettunen et al. (4) reported a relationship between complicated delivery and PPD, especially with pain during delivery. 

Vigod et al. (37) reported in their systematic review that mothers who gave birth to an infant of very preterm or very low birth weight (LBW) had higher levels of depression throughout the first postpartum year. The results of our study showed a significant relationship between infant hospitalization and PPD in mothers who conceived by ART. Mothers in the ART group appeared to worry more about their infants than the control group mothers because of the difficulties with conceiving (infertility, cost of infertility treatment). 

The results of our study showed no significant relationship between occupational and educational status in both groups. Two previous studies have shown that working mothers probably have a protective factor for PPD (38, 39). Lewis et al. (40) observed that employed women reported less symptoms of depression than stay-at-home mothers, regardless of their weekly working hours. Sadr et al. (34) showed that there was no significant relationship between PPD and occupational and educational status.

Limitations of this study included the relatively low sample size in the ART group and limited access to this patient population since some of these women lived in other cities and only referred to Royan Institute for infertility treatment before pregnancy. In addition, we did not have consents from all of the women who became pregnant after ART at Royan Institute and could not enroll them in this study. Further studies with larger sample sizes are recommended. 

## Conclusion

This study reveals that the occurrence of PPD in mothers who conceived naturally is similar to those who conceived via ART. Our study has also provided evidence that levels of education, occupation, type of delivery, history of infant hospitalization, and history of depression are risk factors for PPD in mothers. These factors, rather than conception via ART, should be given further prominence in interventions to prevent PPD in women.
